# Behavior Change Interventions to Address Unhealthy Food Consumption: A Scoping Review

**DOI:** 10.1016/j.cdnut.2024.102104

**Published:** 2024-02-13

**Authors:** Shivani Kachwaha, Sunny S Kim, Jai K Das, Sabrina Rasheed, SubbaRao M Gavaravarapu, Pooja Pandey Rana, Purnima Menon

**Affiliations:** 1 Program in Human Nutrition, Department of International Health, Johns Hopkins Bloomberg School of Public Health, Baltimore, MD, United States; 2 Nutrition, Diets, and Health Unit, International Food Policy Research Institute (IFPRI), Washington, DC, United States; 3 Institute for Global Health and Development, Aga Khan University, Karachi, Pakistan; 4 International Centre for Diarrhoeal Disease Research, Dhaka, Bangladesh; 5 Indian Council of Medical Research-National Institute of Nutrition, Hyderabad, India; 6 Helen Keller International, Kathmandu, Nepal; 7 Food and Nutrition Policy, IFPRI, New Delhi, India

**Keywords:** behavior change interventions, unhealthy food, diet quality, scoping review, food choice

## Abstract

High intakes of sodium, sugar, saturated fats, and trans–fats contributed to 187.7 million disability adjusted life years from noncommunicable diseases globally. Understanding of the global evidence on interventions to reduce consumption of various types of unhealthy food across diverse contexts is needed. We conducted a scoping review to examine the existing evidence on behavior change interventions (BCIs) to address unhealthy food consumption. Through a systematic search of 3 databases conducted in December 2022, 2730 records were retrieved, and 145 studies met the eligibility criteria for review. Only 19% of the studies (*n* = 28) were from low- and middle-income countries. The key target group for most BCIs was adults ≥20 y (*n* = 79). Interventions were conducted across 7 types of settings: schools (*n* = 52), digital (*n* = 30), community (*n* = 28), home (*n* = 14), health facility (*n* = 12), worksite (*n* = 6), and market (*n* = 3). There were 4 mutually inclusive intervention types—information, education, and communication (*n* = 141); food/beverage substitution (*n* = 10); interactive games (*n* = 7); and labeling/warnings at point-of-purchase (*n* = 3). The study outcomes included consumption of sugar-sweetened beverages (*n* = 74), packaged salty snacks/fast food (*n* = 61), sweets (*n* = 43), and saturated fat (*n* = 41). Drivers of food choice behaviors, such as knowledge, attitudes, and beliefs; motivation and expectancies; and self-efficacy were reported in 43% of studies. On the basis of reported impact of BCIs on study outcomes, more interventions targeted at adults had positive impacts compared with those targeted at children; intervention packages, including multiple information, education, and communication components also reported impacts more often than single informational interventions. Interpretation of the findings was complicated by the lack of comparability in interventions, evaluation designs, outcome measures of unhealthy food consumption, duration of interventions, and study contexts. Future studies should invest in critical yet underrepresented regions, examine behavioral determinants of unhealthy food consumption and the sustainability of behavior change, and conduct further analysis of effectiveness from experimental studies.

## Introduction

Modifiable behaviors, including smoking, alcohol abuse, sedentary lifestyle, and poor eating habits [1], increase the risk of noncommunicable diseases (NCDs) such as stroke, diabetes, asthma, chronic obstructive pulmonary disease, and cancers. Populations in low- and lower-middle-income countries (LMICs) are even more susceptible to developing NCDs, given that a high prevalence of undernutrition in early life followed by the nutrition transition increases the risk of NCDs in later life [2]. As such, suboptimal diets are an important preventable risk factor for NCDs. For instance, consuming too much sodium is associated with high-blood pressures, and not enough potassium raises the risk of heart disease and stroke [3]. Intake of high calorie diets with free sugars may lead to unhealthy weight gain, which can subsequently result in overweight and obesity. Dietary risk factors, such as high intakes of sodium, sugar, saturated fats, and trans–fats, contributed to 187.7 million disability adjusted life years from NCDs globally [4]. Global daily consumption of all unhealthy foods, including sugar-sweetened beverages (SSBs—49 g/d), processed meat (4 g/d), sodium (6 g/d), and red meat (27 g/d) exceeded optimal levels [5].

LMICs are facing a major nutrition transition where economic growth has led to industrialization, urbanization, and increased incomes, which in turn are associated with changes in dietary patterns—a shift away from traditional diets (high in whole grains cereals and pulses and low in fat and refined sugars) toward energy-dense, nutrient-poor diets, and processed and ultraprocessed foods (high in saturated fats, salt, and sugar) [1,6]. Efforts to mitigate the negative effects of the nutrition transition include transforming food systems so that they deliver affordable nutritious foods, working with consumers to promote consumption of healthy diets, and adapting space and physical environments to promote and facilitate physical activity. Policy level and food supply side interventions, such as unhealthy food/beverage taxes or warning labels, reformulation of food products (e.g. reduced sodium in bread) or packaging (e.g. reducing portion sizes), and postharvest technologies to introduce nutrient-rich foods for year-round sale in markets at affordable prices could be viable options [[7], [8], [9], [10], [11]]. Policies and programs related to food and diets may also aim to improve the availability, accessibility, and affordability of safe and nutritious foods, and to promote healthy eating habits through education and awareness campaigns.

Behavior change strategies are used to discourage unhealthy food consumption and promote healthy food choices employing techniques, such as nutrition education, goal setting, self-monitoring, and providing feedback as well as support. Behavioral interventions are defined as “coordinated sets of activities designed to change specified behavior patterns” [12]. They are designed to affect the actions and behaviors that individuals take to improve their health outcomes. Behavior change interventions (BCIs) involve several interacting components in their implementation at the individual, household, community, and national levels [12].

Unhealthy food consumption is a complex behavior involving a wide range of unhealthy foods and beverages and drivers associated with both the food environment and individual food choices [13,14], such as cost, convenience, availability, access, physical appeal, addictive taste, and perceived social appeal [15]. Addressing unhealthy food consumption is distinct from promoting healthy food choice alone, in that it requires deterring negative behaviors (often easy, convenient, and appealing) and promoting adoption of positive ones (which may be less tasty or satisfactory).

In LMICs, BCIs have focused primarily on promoting healthy foods, such as fruits and vegetables and on increasing dietary diversity to improve quality of the diet [16,17]. There is an emerging global body of evidence on BCIs to address unhealthy food consumption, but previous scoping and systematic reviews have focused on specific food/beverage or nutrient types such as SSBs or added salt intake alone [[18], [19], [20], [21]]. To the best of our knowledge, there has been no review to date that examined literature on BCIs addressing unhealthy food consumption comprehensively. A landscape of the BCI evidence on unhealthy food consumption is needed to adapt and develop best approaches to address this growing public health and nutrition challenge.

The primary objective of this scoping review was to assess the size and scope of the current global evidence on BCIs addressing consumption of unhealthy foods and to highlight areas for further inquiry. The secondary objectives of the review were to examine the drivers of food choice that are addressed by BCIs targeting unhealthy food consumption and reported impacts of BCI interventions on unhealthy food consumption outcomes.

## Methods

### Study design

This study is designed as a systematic scoping review to assess the size and scope of available global literature on BCIs for addressing a range of outcomes related to unhealthy food consumption. The scoping review followed the “PRISMA Protocols Extension for Scoping Reviews” guidelines in reporting study procedures and findings [22].

### Definition of unhealthy foods

For the scoping review, unhealthy foods were operationally defined as specific foods/beverages, such as SSBs, sweets, processed meat, refined grains, deep-fried foods, added salt, and added sugar; and nutrients, such as saturated and trans-fat, and cholesterol. Diets characterized by high levels of these foods and low in whole grains, fruit, and vegetables are considered unhealthy [23]. For terms and definitions used in our review, we referred to diet quality indices that classify and measure unhealthy food consumption with 3 major categories, including nutrient-based indicators, food or food group-based indicators, and combination indices (Supplemental Table 1) [7]. We purposively adopted an expansive definition of unhealthy foods and nutrients to search for and identify all studies that address a wide range of unhealthy food consumption outcomes.

### Drivers of food choice

Consumption is a behavior influenced by various determinants of food choice, and behavioral interventions address one or more of these determinants as intermediate outcomes along the dietary intake pathway. During data extraction of study records, we applied the 24 constructs of food choice drivers developed by the Drivers of Food Choice Program at the University of South Carolina, categorized as interpersonal, sociocultural, personal food environment, material assets and resources, and person-state drivers (Supplemental Figure 1). These constructs were developed based on an extensive review of existing tools and resources for measuring drivers of food choice [24]. We examined which food choice drivers were targeted or addressed by behavioral interventions where such data were available.

### Literature search strategy

The literature search was conducted in 3 databases across life sciences and biomedical/health topics on social sciences—PubMed, Epistemonikos, and Web of Science—for articles published between January 2000 and December 2022. Search terms were developed based on the operational definitions of unhealthy foods and aspects of consumption, study type, and behavior change (Supplemental Table 2). Search results were uploaded into Endnote, duplicates removed, and then imported to Covidence (web-based screening and data extraction tool) [25]. The literature search was completed on December 12, 2022.

### Eligibility criteria

We included randomized controlled trials, quasi-experimental (pre-post or nonrandomized studies), and mixed methods studies assessing BCIs that addressed consumption of unhealthy food(s) directly among consumers or beneficiaries, and reporting outcomes for the consumption of unhealthy food. We only included studies conducted on humans published as peer-reviewed articles where full texts were available in English. We excluded studies without a BCI and not reporting specific outcomes on unhealthy food consumption. We excluded clinical studies (studies on treatments/therapies), cross-sectional/observational studies, or studies applying qualitative methods only. We also excluded studies solely targeting high-risk populations with chronic conditions, such as obesity/overweight, kidney disease, cancer, heart disease, autism, etc., because of their limited generalizability. These studies typically had specialized interventions targeting chronic conditions, and high-risk population groups may have a greater risk perception and motivation to change their behaviors as compared with those without these conditions. Lastly, we excluded studies on food/beverage tax and subsidies and food labeling policies, because they were focused on broader policy instruments for the general population and not individual consumers.

### Screening and data extraction

The first reviewer (SK) reviewed titles, abstracts, and full texts for articles. A second reviewer (FS) supported in reviewing full texts for all articles to assess for study inclusion. Both reviewers extracted data for a separate set of studies, and the 2 reviewers met regularly to resolve conflicts on full texts to be included, with support from the senior investigator (SSK) to ensure that data were extracted according to the study protocol. Data were extracted based on a structured template developed in Covidence, adapted from the Population, Intervention, Comparator, Outcome protocol [26]. The template included study information on author, year, location, population [preschool-aged children (0–5 y), older children or adolescents (6–19 y), or adults (≥20 y)], sample size, study design (randomized controlled trials, quasi-experimental—pre-post/nonrandomized studies, or mixed methods studies), drivers of food choice, unhealthy food consumption outcomes, intervention details/BCI components, and significance of findings. Information on geographic location was extracted based on World Bank classification of regions and high-/low-income countries [27].

### Data synthesis and analysis

We identified 9 categories of unhealthy foods based on the definitions above and informed by specific foods/beverages or nutrients as reported in the studies. The categories included the following: *1*) SSBs and sodas; *2*) packaged salty snacks and fast food (chips, pizza, burgers, etc.); *3*) sweets (ice cream, candy, chocolates, cakes, cookies etc.); *4*) fried food (fried potatoes or vegetables, and fried dough); *5*) refined grains; *6*) processed meat; *7*) added table salt/sodium; *8*) saturated fat/trans-fat; and *9*) cholesterol. We also extracted intermediate outcomes on any of the 24 drivers of food choice (described above).

Intervention duration was defined as the time period (in weeks, months, or years) that study participants were exposed to the BCIs as reported in the studies. Interventions were categorized by delivery platforms or 7 settings: school, home, community, digital, health facility, worksite, and market. Schools included preschool, middle or high school, college, or university. Communities included different types of community gatherings, centers, or facilities. Digital settings included telephone, e-mail, text, or interactive applications. Health facilities included clinics or hospitals. Worksites included specific companies or industrial workplaces. Markets included stores, vendors, and any retail places where food and beverages are sold.

Intervention types were categorized as information, education, and communication (IEC); food/beverage substitution; menu/food labeling/warnings at point-of-purchase; and interactive games. IEC interventions were further grouped as message dissemination or informational; individual or group counseling and nutrition education; and mass media strategies, such as radio, television, and advertisements. Food/beverage substitution interventions included provision of specific foods (e.g., milk) with the intention of replacing unhealthy foods/beverages, such as SSBs. Labeling/warnings at point-of-purchase included interventions that used printed health warnings or labeling of nutrition information with an intent to deter purchase and consumption of specific unhealthy foods or beverages (e.g., warnings on high sugar content and health consequences of SSBs). Interactive games included nutrition messages/information provided through fun activities, such as card games or computer games.

Extracted information on target groups, duration, sample size, intervention types, BCI components, and study outcomes on unhealthy food consumption were summarized by intervention setting. Impacts on unhealthy food outcomes reported in the studies were classified as having either positive significant impact or null impact for each outcome, and the proportion of studies in either category was used to make comparisons. Effect sizes were not analyzed and/or compared, as study designs, outcome indicators, and measurement methods varied. We conducted subgroup analysis to examine the impacts reported on selected outcomes by target groups and intervention types.

## Results

### Description of studies

**FIGURE 1:**
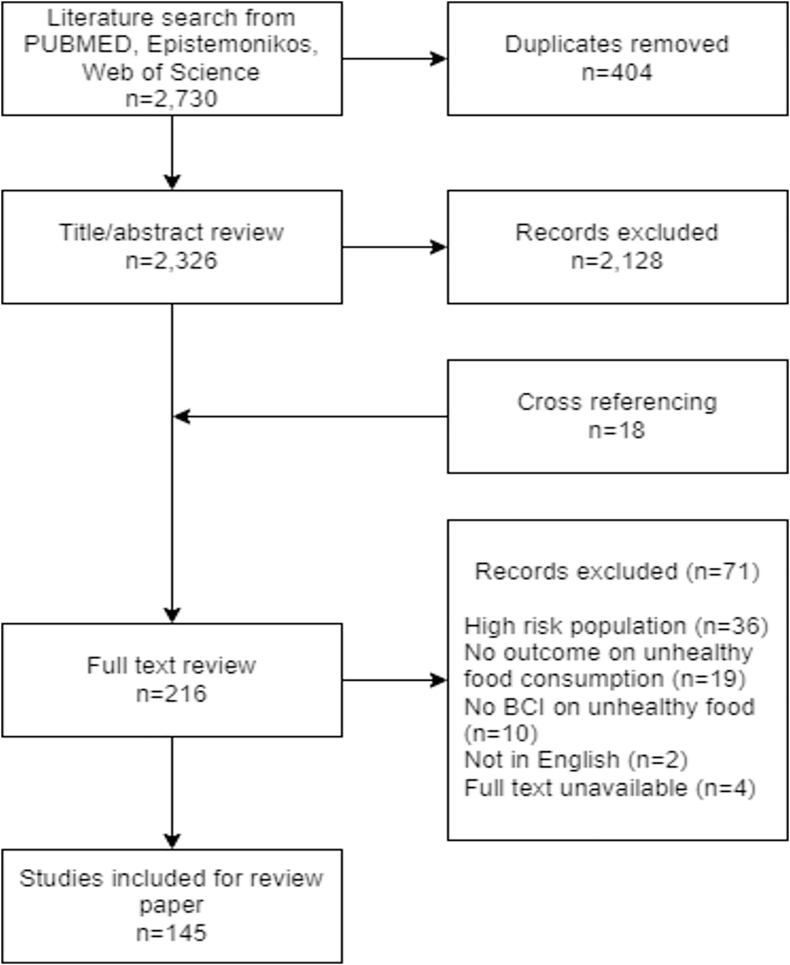
Study flow diagram.

The literature search retrieved 2730 articles across the 3 databases (Figure 1). Titles and abstracts were reviewed for 2326 to determine eligibility after excluding 404 duplicates. After cross-referencing (*n* = 18), a total of 216 articles were considered for full-text review. During the full-text review, 71 articles were excluded based on the exclusion criteria with the most frequent reasons being high-risk population (*n* = 36) or no specified outcomes on unhealthy food consumption (*n* = 19). Finally, 145 articles of unique studies met the eligibility criteria and were included in the scoping review, with data extracted based on the template described above (Supplemental Table 3).

Most studies were from high-income countries (*n* = 117, 80.7%), with the majority from North America (*n* = 55) and Europe (*n* = 43) (Table 1). Only 28 studies (19.3%) were from LMICs, and among them, 12 were from Latin America, 5 each from South Asia and Middle East/North Africa, 2 from Sub-Saharan Africa, and 4 from Central/East Asia. Most of the studies were conducted in urban areas only (*n* = 129, 89%), 11 studies were conducted in rural areas only, and 5 studies were in both urban and rural areas.

**TABLE 1:** Summary of studies included in the scoping review

Category	Number of studies (*N* = 145) *n* (%)
Income group	
High-income country	117 (80.7)
Low- to middle-income country	28 (19.3)
Region	
South Asia	5 (3.4)
Latin America and Caribbean	12 (8.3)
Middle East and North Africa	5 (3.4)
North America	55 (37.9)
Europe and Central Asia	43 (29.7)
East Asia and Pacific	23 (15.9)
Sub-Saharan Africa	2 (1.4)
Location	
Urban	129 (89.0)
Rural	11 (7.6)
Urban and rural (mixed)	5 (3.4)
Target group^1^	
Young children (0–5 y)	19 (11.4)
Older children/adolescents (6–19 y)	68 (41.0)
Adults (≥20 y)	79 (47.6)
Study design	
Randomized controlled trial	93 (64.1)
Quasiexperimental	48 (33.1)
Mixed methods	4 (2.8)
Intervention setting	
School	52 (35.9)
Home	14 (9.7)
Community	28 (19.3)
Digital	30 (20.7)
Health facility	12 (8.3)
Worksite	6 (4.1)
Market	3 (2.1)
Intervention type^1^	
Information, education, and communication (IEC)^2^	141 (87.6)
Messages or information (texts, e-mails, print materials, etc.)	65 (46.1)
Individual/group counseling (education, coaching)	105 (74.5)
Mass media approaches (radio, TV, advertisements, etc.)	8 (5.7)
Multiple IEC component interventions	36 (25.5)
Food/beverage substitution	10 (6.2)
Labeling/warnings at point-of-purchase	3 (1.9)
Interactive games	7 (4.3)
Studies reported drivers of food choice behaviors^1^	62 (42.8)
Outcomes on unhealthy food consumption^1^	
Sugar-sweetened beverages/sodas (SSBs)	74 (25.3)
Packaged salty snacks/fast food (chips, pizza, burgers, etc.)^3^	61 (20.7)
Sweets (ice cream, candy, chocolates, cakes, cookies, etc.)	43 (14.7)
Fried food (fried potatoes or vegetables, fried dough, etc.)	17 (5.8)
Refined grains	5 (1.7)
Processed meat	15 (5.1)
Added table salt/sodium	29 (9.9)
Saturated fat/trans-fat	41 (14.0)
Cholesterol	9 (3.1)

Abbreviations: IEC, information, education, and communication; SSB, sugar-sweetened beverage.

1 Target group, intervention types, and study outcomes were inclusive and overlapping categories, so total numbers exceed number of studies in review (145).

2 IEC intervention types included messages or information (targeted to specific individuals or groups in study), counseling, and mass media (targeted to general population). Multiple IEC component interventions included combinations of information, counseling, and/or mass media intervention types.

3 These studies classified snacks/fast foods generally and did not distinguish specific types.

The key target groups were adults aged ≥20 y (*n* = 79, 47.6%) and older children/adolescents (*n* = 68, 41%), with few studies targeted to pre-school-aged children <6 y (*n* = 19, 11.4%). Most studies were randomized controlled trials (*n* = 93, 64.1%); 48 studies (33.1%) used quasi-experimental designs, and 4 studies (2.8%) used mixed methods.

The most common intervention settings were schools (*n* = 52, 35.9%), digital platforms (*n* = 30, 20.7%), and community (*n* = 28, 19.3%) (Table 1). Nearly all studies involved IEC interventions (*n* = 141, 87.6%), and among them, 105 provided individual or group counseling, 65 provided messages or information, and only 8 used mass media. Mass media interventions included information disseminated to the general population, using different media strategies, such as television, radio, advertisements, etc. A total of 71 studies provided counseling only, 31 provided information only, and 36 included multiple IEC components (information and counseling; counseling and mass media; information and mass media; or information, counseling, and mass media). Other intervention types were food/beverage substitution (*n* = 10, 6.2%), interactive games (*n* = 7, 4.3%), and labeling/warnings at point-of-purchase (*n* = 3, 1.9%).

The outcomes on unhealthy food consumption most frequently reported were SSBs (*n* = 74, 25.3%), packaged salty snacks and fast food (*n* = 61, 20.7%), sweets (*n* = 43, 14.7%), and saturated fat (*n* = 41, 14%) (Table 1). Other outcomes less reported included added table salt (*n* = 29, 9.9%), deep-fried food (*n* = 17, 5.8%), processed meat (*n* = 15, 5.1%), cholesterol (*n* = 9, 3.1%), and refined grains (*n* = 5, 1.7%). Approximately 42.8% of studies (*n* = 62) reported on any drivers of food choice.

### Interventions and settings

The intervention elements, such as target group, duration, sample size, intervention components, and outcomes, varied by intervention setting. Among the 52 school interventions, 42 targeted older children and adolescents and ranged widely in duration (10 days to 6 years) and sample size (ranging from 34–3500 participants) (Table 2). Home interventions (*n* = 14) were targeted to adults (*n* = 9) and older children (*n* = 6), had intervention durations of 1 month to 2 years, and sample sizes of 58–880 across the studies. Most community interventions (*n* = 28) were targeted to adults (*n* = 23), lasted 7 days to 2 years, and included 36–1903 study participants.

**TABLE 2:** Summary of intervention elements by setting (*n* = 145)

Intervention setting	Target group	Duration	Sample size	Intervention types	Intervention components	Unhealthy food consumption outcomes
School (*n* = 52)	Young children: 3 studies Older children: 42 studies Adults: 9 studies	10 days to 6 years	34–3500	IEC: 51 studies - Counseling: 38 studies - Information: 21 studies - Mass media: 3 studies Food/beverage substitution: 3 studies Interactive games: 3 studies	- Group nutrition counseling provided in school/classroom settings - Focus on simple/easy to understand messages to increase awareness on the risks of specific unhealthy foods - Counseling by trained teachers/peer educators/program staff - Provision of BCC materials such as booklets, newsletters, fact sheets, etc.	- SSBs: 34 studies - Packaged salty snacks/fast food: 28 studies - Sweets: 12 studies - Saturated fat: 8 studies
Home (*n* = 14)	Young children: 3 studies Older children: 6 studies Adults: 9 studies	1 month to 2 years	58–880	IEC: 14 studies - Counseling: 12 studies - Information: 9 studies Food/beverage substitution: 1 study	- Individual counseling provided to household members (children, mothers) - Messages reiterated through regular home visits or follow-up calls - Provision of materials such as weekly handouts, newsletters, etc.	- SSBs: 7 studies - Packaged snacks/fast food: 7 studies - Sweets: 4 studies - Added salt: 4 studies
Community (*n* = 28)	Young children: 4 studies Older children: 7 studies Adults: 23 studies	7 days to 2 years	36–1903	IEC: 28 studies - Counseling: 23 studies - Information: 12 studies - Mass media: 3 studies Food/beverage substitution: 3 study Interactive games: 1 study	- Group-based counseling and workshops held at community centers, recreational centers, or designated childcare centers - Messages focused on consequences of unhealthy food and resources/strategies to reduce consumption - Focus on feedback, reinforcement, goal setting, and skill building - Distribution of brochures, pamphlets, tool kits etc.	- SSBs: 16 studies - Packaged snacks/fast food: 11 studies - Sweets: 7 studies - Added salt: 11 studies
Digital (*n* = 30)	Young children: 3 studies Older children: 8 studies Adults: 22 studies	7 days to 1 year	30–2159	IEC: 28 studies - Counseling: 16 studies - Information: 15 studies - Mass media: 1 study Food/beverage substitution: 2 studies Interactive games: 2 studies	- Information or messages provided via texts, e-mail, interactive apps, phone calls, etc. - Focus on personalized feedback, goal setting, and self-monitoring	- SSBs: 11 studies - Packaged snacks/fast food: 10 studies - Sweets: 13 studies - Added salt: 11 studies
Health facility (*n* = 12)	Young children: 5 studies Older children: 4 studies Adults: 8 studies	4 weeks to 6 years	70–1062	IEC: 12 studies - Counseling: 10 studies - Information: 3 studies	- Individual counseling at primary health clinics or hospitals by health professionals/dietitians - Focus on providing personalized goals or diet plans to reduce unhealthy food consumption	- SSBs: 4 studies - Packaged snacks/fast food: 3 studies - Sweets: 4 studies - Saturated fat: 7 studies
Worksite (*n* = 6)	Adults: 6 studies	8 weeks to 1 year	53–850	IEC: 6 studies - Counseling: 4 studies - Information: 4 studies Food/beverage substitution: 1 study	- Group counseling at workplace sites - Materials included handouts, weekly newsletters	- Sweets: 2 studies - Added salt: 2 studies - Saturated fat: 4 studies - Cholesterol: 3 studies
Market (*n* = 3)	Older children: 1 study Adults: 2 studies	3–8 months	74–1003	IEC: 2 studies - Counseling: 1 study - Information: 1 study - Mass media: 1 study Labeling/warnings at point-of-purchase: 3 studies	- Labeling on unhealthy foods with nutrient and calorie information - Nutrition information provided at point-of-sale - Counseling on reading nutrition labels	- Added salt: 2 studies - Saturated fat: 2 studies - SSBs: 1 study

Target group, intervention types, and all study outcomes were inclusive fields, so total numbers exceed number of studies.

Abbreviations: BCC, Behavior change communication; IEC, information, education, and communication; SSB: sugar-sweetened beverage.

Among the 30 digital interventions, 22 targeted adults, with intervention durations of 7 days to 1 year, and sample size of 30–2159 (Table 2). Most health facility interventions (*n* = 12) also targeted adults (*n* = 8), lasting from 4 wk to 6 y, and included 70–1062 study participants. Finally, both worksite (*n* = 6) and market interventions (*n* = 3) focused on adults and had shorter intervention durations of 8 weeks to 1 year and 3–8 months, respectively, with sample sizes ranging from 53 to 1003 participants.

For intervention types across all settings, IEC was the most common (*n* = 141), except for market settings that mostly used labeling/warnings at point-of-purchase (Table 2). Individual/group counseling was most often used in school, home, community, and health facility settings. Digital and worksite interventions applied both counseling- and information-focused IEC.

Intervention activities at school settings included nutrition counseling provided by trained teachers/peer educators in classrooms (Table 2). Home interventions typically involved individual counseling provided to different family members and follow-up home visits, whereas community interventions focused on group-based counseling. Digital interventions utilized texts, e-mails, and interactive applications to provide messages and personalized feedback, with an emphasis on self-monitoring. Health facility interventions included individual counseling and provision of specific diet plans with goal setting. Worksite interventions included group counseling, and market interventions provided nutrition information and health warnings at point-of-sale as well as labeling of unhealthy foods. All interventions applied print materials, such as handouts, newsletters, fact sheets, and tool kits to promote and reinforce nutrition messages.

The main outcomes on unhealthy food consumption reported in school interventions were SSBs (*n* = 34) and packaged salty snacks/fast food (*n* = 28) (Table 2). Home and community interventions also often reported outcomes on SSBs (*n* = 7 and *n* = 7, respectively) and packaged snacks (*n* = 16 and *n* = 11, respectively). Digital interventions mostly addressed consumption of sweets (*n* = 13), followed by SSBs (*n* = 11) and packaged snacks (*n* = 10). Health facility, worksite, and market interventions reported most frequently on saturated fat intake (*n* = 7, 4, and 2, respectively).

### Drivers of food choice

One or more drivers of food choice were explicitly measured and reported in 62 studies (42.8%) (Table 3). The most common drivers reported with high frequency were knowledge, attitudes, and beliefs (*n* = 38) and motivation and expectancies (*n* = 21). Other drivers reported included self-efficacy (*n* = 12), preferences (*n* = 7), habits and routines (*n* = 7), and social relationships (*n* = 8). Few studies reported on addressing identity (*n* = 1), goals and prioritization (*n* = 3), values (*n* = 1), affordability (*n* = 4), accessibility (*n* = 2), wealth (*n* = 4), and biological features (*n* = 1). Drivers not addressed in any of the studies were gender, life course perspectives, food and water security, and desirability.

**TABLE 3:**
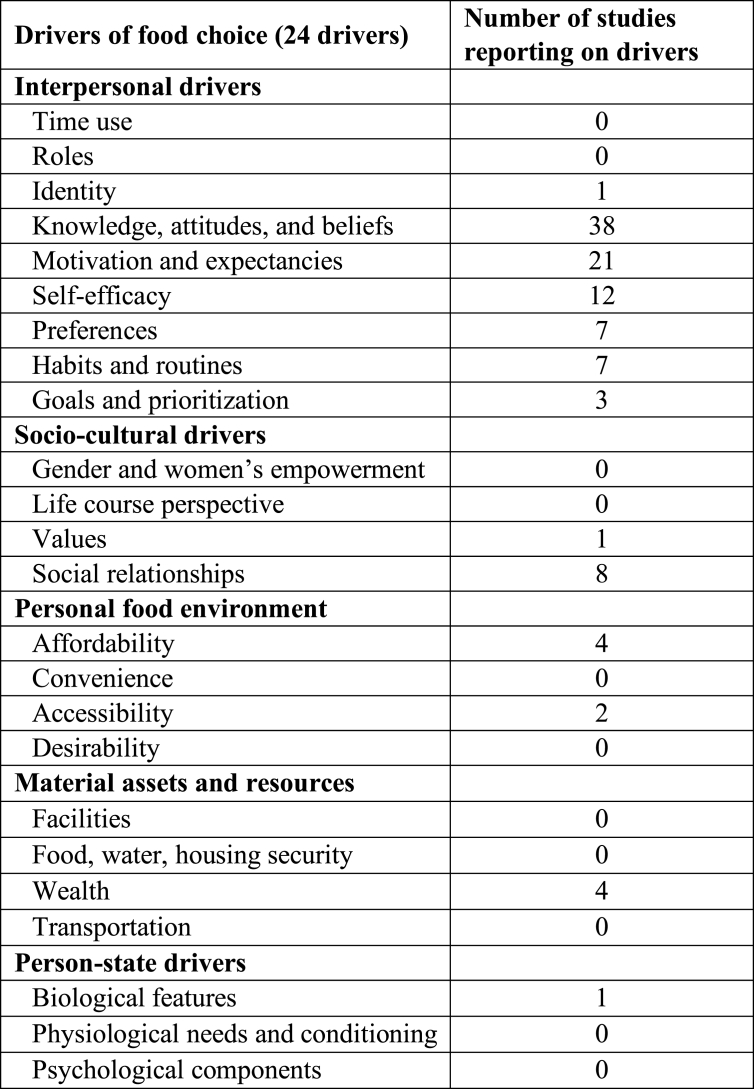
Drivers of food choice reported in intervention studies (*n* = 62)

### Impact of BCIs

**FIGURE 2:**
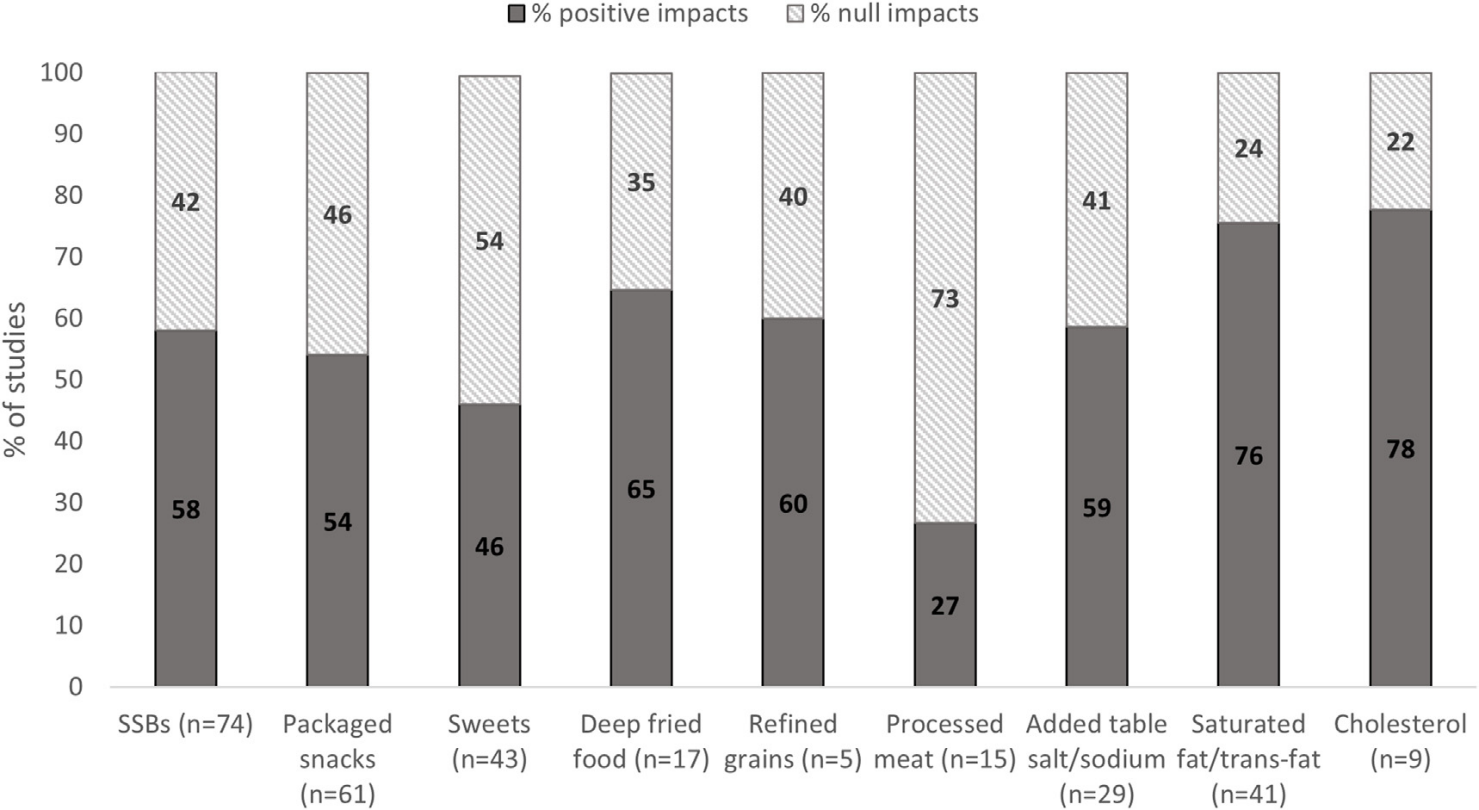
Percentage of studies reporting impact by unhealthy food consumption outcome (*n* = 145). SSB, sugar-sweetened beverage.

**FIGURE 3:**
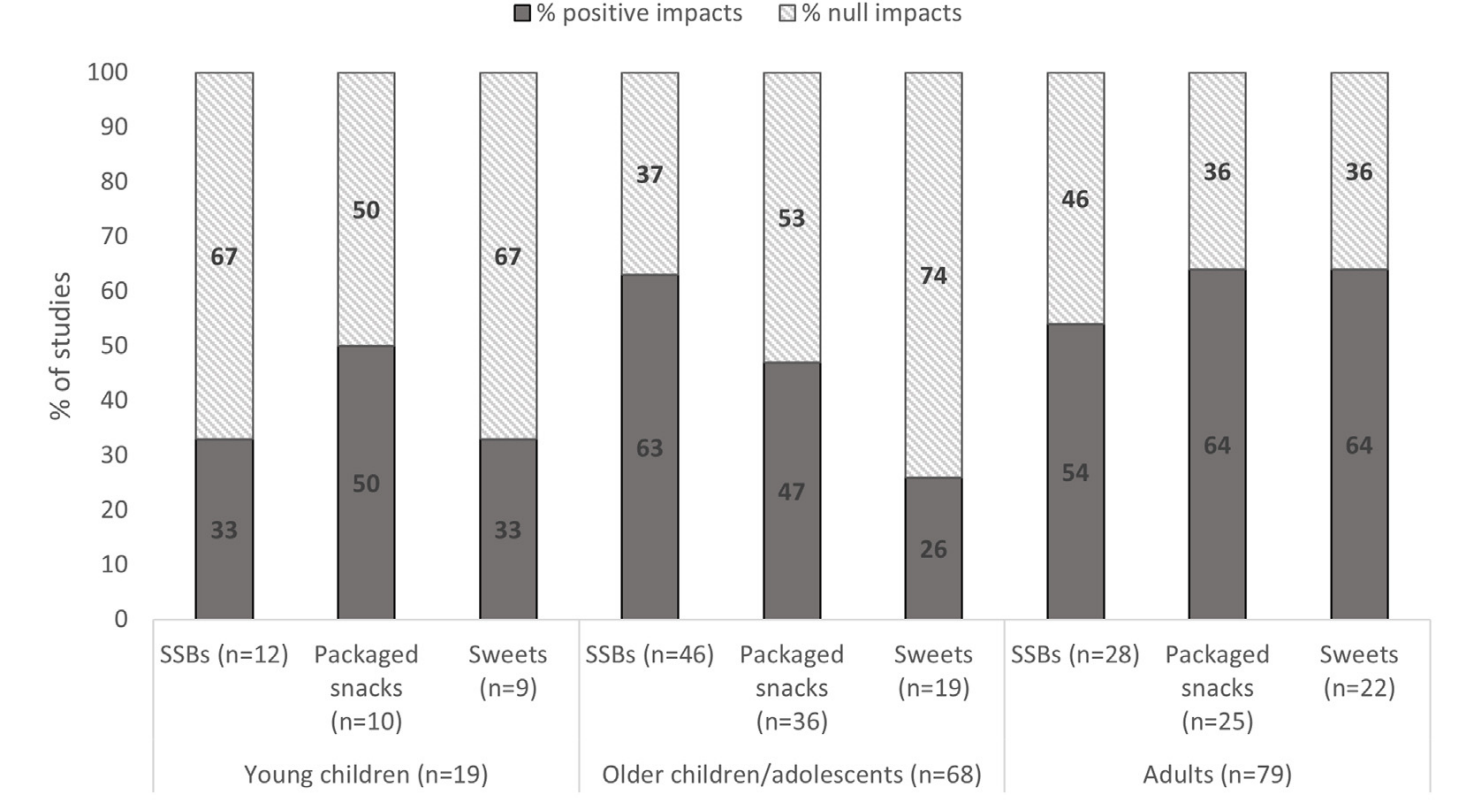
Percentage of studies reporting impact on main outcomes of unhealthy food consumption by target group (*n* = 145). SSB, sugar-sweetened beverage.

**FIGURE 4:**
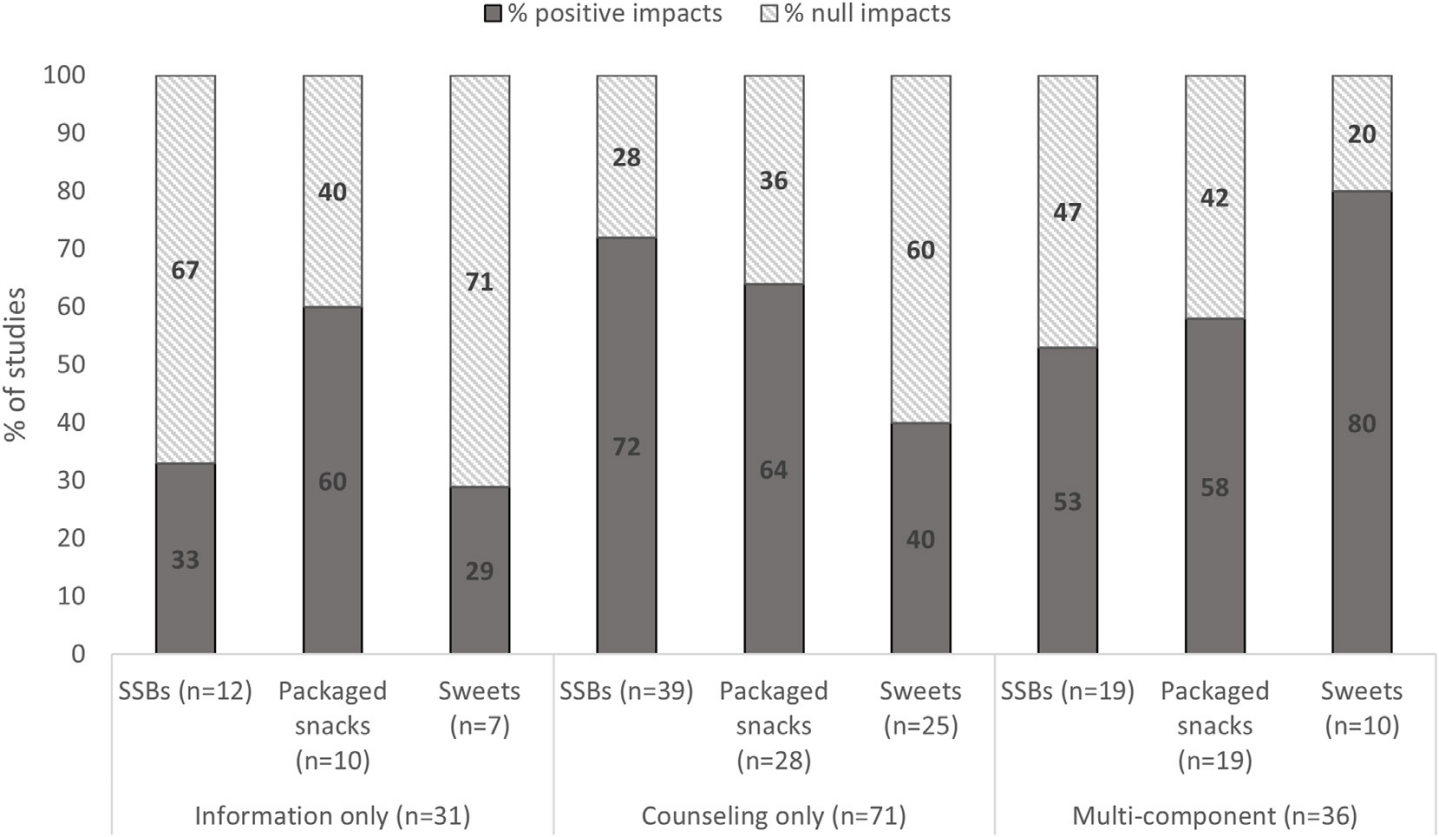
Percentage of studies reporting impact on main outcomes of unhealthy food consumption by IEC intervention type (*n* = 141). Multicomponent interventions include information, counseling, and/or mass media intervention types. IEC, information, education, and communication; SSB, sugar-sweetened beverage.

Mixed results were observed for reported impacts of BCIs on unhealthy food consumption outcomes (Figure 2). More than half of the studies (among those who measured these outcomes) reported impacts in reducing the consumption of saturated fat (76%), added table salt (59%), SSBs (58%), and packaged salty snacks/fast food (54%). Null impacts were reported for most studies targeting processed meat (73%) and sweets (54%). For the 3 most common outcomes (SSBs *n* = 74, packaged snacks *n* = 61, and sweets *n* = 43), we observed that higher proportion of interventions targeting adults reported impacts compared with those targeting young children or older children/adolescents (Figure 3). In addition, more studies that applied counseling and multiple IEC component interventions (counseling, information and/or mass media) reported positive impacts compared with those providing information alone (Figure 4). When comparing reported impacts by high-income countries and LMICs, we observed that a higher proportion of studies in high-income countries (HICs) found positive impact on consumption of sweets compared with LMICs, and the opposite trend was observed for SSB consumption (Supplemental Figure 2).

## Discussion

The 145 studies included in this scoping review highlighted the growing body of evidence on BCIs to address unhealthy food consumption. Published literature was concentrated in HICs, urban settings, and focused largely on adolescents and young adults. A small proportion of studies were available from LMICs and rural contexts. The study design, target group, intervention duration, BCI components, and outcomes varied extensively. Less than half of the published literature reported on intermediary drivers of unhealthy food choices and consumption behavior, limiting our understanding of how impacts on behavior change was/was not achieved.

A critical gap identified by this review was the scarce evidence from LMICs. Regions such as South Asia, Latin America, and Sub-Saharan Africa are facing high burdens of malnutrition, rising NCDs, and ongoing nutrition transition characterized by rapidly changing food environments, but evidence from these areas are highly underrepresented. Investments in more studies in these contexts are essential for creating a stronger evidence base for action. Similarly, studies on rural contexts were underrepresented in our review, which is an important focus area for future studies. In addition, few studies included young children as a target group, which may be a key population group to inculcate healthy eating behaviors early in life.

Another gap was the lack of reporting on the key behavioral determinants addressed by the behavioral interventions. Among the few studies that reported on food choice drivers, most concentrated on knowledge, attitudes, beliefs, and motivation. The importance of focusing on drivers at different levels including sociocultural and personal food environment for improved health behaviors and dietary outcomes is well recognized in the literature [28,29]. For example, previous studies have highlighted that improved health behaviors are strongly associated with improved knowledge, motivation, and self-efficacy [30]. Understanding which and how key drivers are addressed by behavioral interventions would help to explain why certain studies achieved impact or not. Studies to assess the impacts of BCIs should consider measuring and reporting on drivers of food choices to understand the mechanisms of behavior change to reduce unhealthy food consumption.

Our scoping review included a broad set of outcomes related to unhealthy food/beverage and nutrient consumption, compared with other systematic and scoping reviews to date that have focused on a single type of food or nutrient. For example, a series of reviews have been conducted specifically focusing on interventions targeting SSB consumption [18,19,31,32] and added salt consumption [20,21,33,34]. A meta-analysis of 40 studies examining the impact of behavior change techniques for reducing SSB consumption found positive outcomes from interventions targeting children and adolescents, but not for adults [18]. In our review, however, we observed that a higher number of studies on interventions on SSB consumption targeting adults and older children/adolescents reported a positive effect compared with those targeting young children. This could be attributed to differences in study design, time period, duration of intervention, and sample size. The said meta-analysis included a smaller sample (*n* = 40) of randomized or nonrandomized controlled trials published between 1990 and 2016 [18], whereas our review included a larger sample of studies (*n* = 74) across a broader range of designs from the year 2000 onward. Furthermore, it should be noted that this scoping review aimed to examine general patterns rather than comparing effect sizes. Another review of 36 studies demonstrated that behavioral interventions alone or interventions combining behavioral/educational and legislative/environmental approaches were almost equally effective in reducing SSB consumption [31]. Although our review did not focus on legislative or environmental approaches, we observed that more multicomponent IEC interventions reported positive impact compared with single component interventions. It should be noted that most previous reviews considered different types of interventions rather than focusing on BCIs specifically.

On the basis of our comparison of reported impacts across study outcomes and subgroup analysis by target group intervention and type, we observed several prominent patterns. More than half of the interventions targeting adults reported positive impacts on consumption of packaged salty snacks/fast food and sweets, whereas less than half of interventions targeting young children or adolescents reported impacts. Potential reasons why interventions targeting adults may have been more successful include greater cognitive ability, risk perceptions, motivation to change behaviors, and self-efficacy as compared with children [35]. We also observed that substantially more studies targeting certain outcomes, such as processed meat consumption reported null impacts, which could imply that consumption of some unhealthy foods may be particularly challenging to change [36].

In addition, a higher proportion of IEC interventions that included multiple components of information/messaging, counseling, and/or mass media strategies reported impacts compared with interventions providing information only. Approximately 60%–80% of multicomponent IEC interventions reported impacts on consumption of SSBs, packaged salty snacks and fast food, and sweets, compared with interventions with information only. This suggests that interventions including different types of complementary BCI strategies or multiple platforms may be more successful at addressing outcomes of unhealthy food compared with interventions with a single component of message delivery [37]. We note that differences in findings across outcomes may be explained by multiple intervention factors, including study duration, intensity, and intervention types. In addition, changes in unhealthy food consumption were primarily reported through self-reported food frequency questionnaires, 24-h recalls, or food diaries, which have limitations for objectively measuring dietary intake.

Lastly, very few studies in our review assessed the retention and sustainability of behavior changes after the completion of interventions. In the absence of such information, it is difficult to assess if the reported reductions achieved in consumption of unhealthy foods are short-term dietary changes or long-term and sustained behavior changes [38].

Our review had a few limitations. We did not include policy interventions, such as food/beverage taxation and subsidies or other interventions not targeted to individuals. We did not focus on interventions for a single food/beverage or nutrient or effect sizes for reasons outlined in our methodology. The lack of consistency and comparability in measurement of outcomes across studies may have constrained our empirical conclusions about the effectiveness of different types of BCIs to address unhealthy food consumption.

This scoping review identified the growing body of evidence and gaps in the current evidence on BCIs for addressing unhealthy food consumption, particularly the limited evidence from LMICs. Key takeaways from this review include the need to emphasize focus on underrepresented regions in LMICs and rural areas, population groups, such as young children, and behavioral determinants of unhealthy food consumption, including drivers of food choice. Future research may include empirical analysis of effectiveness on addressing unhealthy food consumption as well as examining effects on health outcomes. Future studies and reviews may also focus on examining the duration and sustainability of BCIs addressing unhealthy food consumption.

## Author contributions

The authors’ responsibilities were as follows – SK, SSK, PM: conceived the paper; SK, SSK, JKD, SR, SMG, PPR: developed the scoping review protocol; SK: conducted the systematic search and screening; SK, SSK: interpreted the data and prepared the first draft of the manuscript; JKD, SR, SMG, PPR, PM: contributed to data interpretation and writing of the manuscript; and all authors: read and approved the final manuscript.

## Conflict of interest

The authors report no conflicts of interest.

## Funding

This study was supported by the CGIAR Trust Fund (https://www.cgiar.org/funders).
