# Supplementary material for: Behavior Change Interventions to Address Unhealthy Food Consumption: A Scoping Review
Source: Curr Dev Nutr. 2024 Feb 13;8(3):102104. doi: 10.1016/j.cdnut.2024.102104 (PMC10933472; doi:10.1016/j.cdnut.2024.102104)
Supplement: Multimedia component 1 [file mmc1.docx]

**SUPPLEMENTARY MATERIALS: FOR PUBLICATION**

**Supplemental Table 1: Description of diet quality indices and their definitions of unhealthy foods**

| **Index** | **Description** | **Unhealthy food definition** |
| --- | --- | --- |
| Diet quality index-international (DQI-I) | The DQI-I focuses on four aspects of diet: variety, adequacy, moderation, and balance. Moderation evaluates intake of food and nutrients related to chronic diseases needing restriction. | Saturated fat, cholesterol, and sodium and foods low in nutrient density (table sugar, oil). |
| Healthy Eating Index (HEI) | The HEI-2015 includes separate components for added sugars and saturated fats based on the Dietary Guideline for Americans to limit intakes to <10% of energy. | Intake of sugars and saturated fats contributing to >10% of energy intake. Sodium intake. |
| Global diet quality score (GDQS) | The GDQS score includes 7 unhealthy food groups and 2 food groups classified as unhealthy when consumed in excessive amounts. | Unhealthy: processed meat, refined grains, sugar sweetened beverages, sweets and ice cream, juice, white roots and tubers, purchased deep fried foods.  Unhealthy in excess: high fat dairy, red meat. |
| NOVA classification | NOVA classifies foods according to the extent of processing (unprocessed, processed culinary, processed, and ultra-processed foods). Ultra-processed foods are formulations of cheap industrial ingredients plus additives. | Soft drinks, packaged snacks, re-constituted meat products, and frozen dishes. Foods that are energy-dense, high in unhealthy fats, refined starches, free sugars and salt, and low in nutrient density (“junk food”). |

**Supplemental Table 2: Scoping review search strategy**

PubMed, Epistemonikos, and Web of Science databases were searched with variations of the following *search terms*:

| **Search area** | **Search term** |
| --- | --- |
| Unhealthy foods/ nutrients | ("Sugar-Sweetened Beverages"[Mesh] OR "Fast Foods"[Mesh] OR "unhealthy food*" OR "fried food*" OR "junk food*" OR "Snacks"[Mesh] OR "processed meat" OR "ultra-processed" OR "refined grains" OR “Candy”[Mesh] OR "Sodium, Dietary"[Mesh] OR “Dietary Sucrose”[Mesh] OR "Diet, High-Fat"[Mesh] OR "saturated fat" OR "Trans Fatty Acids"[Mesh] OR "Cholesterol, Dietary"[Mesh]) |
|  | AND |
| Consumption | ("Eating"[Mesh] OR “intake” OR “consum*”) |
|  | AND |
| Study type | ("intervention" OR "program" OR “strateg*”) |
|  | AND |
| Behavior change | ("behavior change" OR "Habits"[Mesh] OR "Counseling"[Mesh] OR “practice*” OR "nutrition education”) |

**Supplemental Figure 1: Drivers of Food Choice constructs**


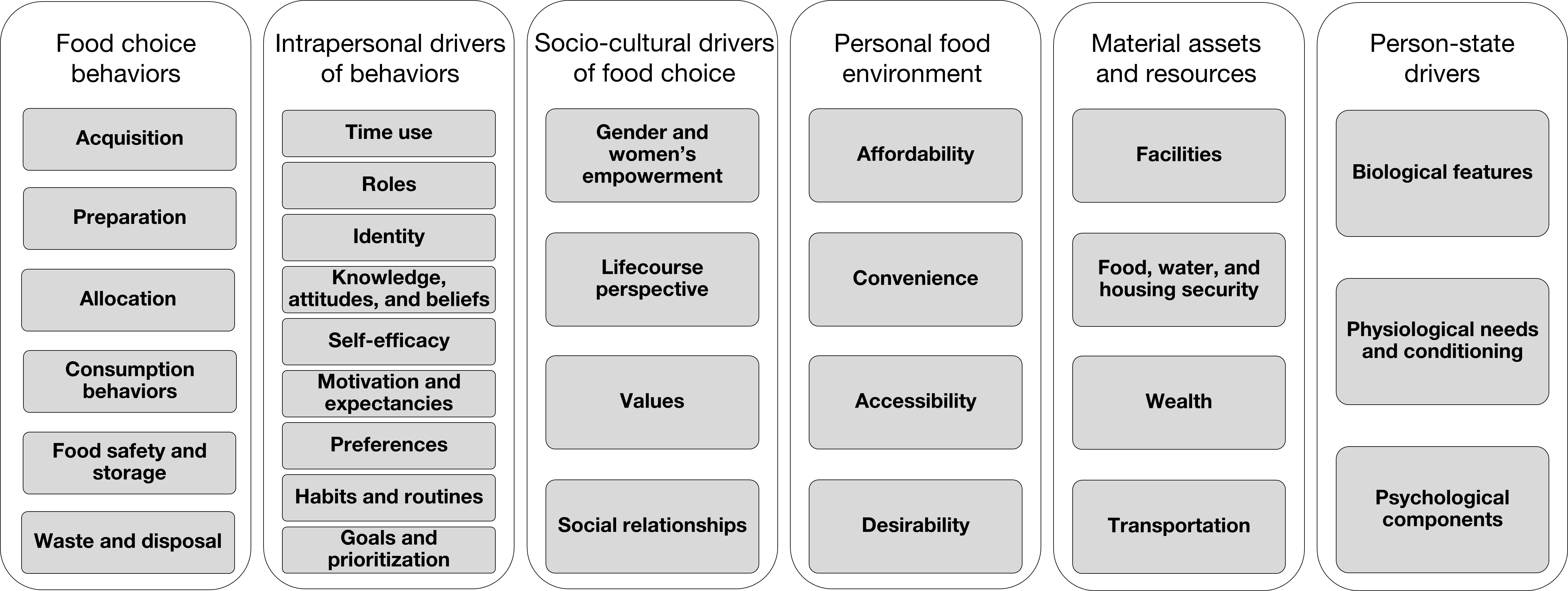


Reference: University of South Carolina, Drivers of Food Choice methods tool repository (2023)

**Supplemental Figure 2: Percentage of studies reporting impact on main outcomes of unhealthy food consumption by high income or low-middle income country status (n=145)**

Note: SSBs- sugar sweeetened beverages, HICs- high-income countries, LMICs- low-middle income countries

**Supplemental Table 3: List of studies included in scoping review, by region and target group (n=145)**

| **S. No.** | **Study title** | **Author** | **Year** | **Region** | **Target group** |
| --- | --- | --- | --- | --- | --- |
|  | School-based intervention to enable school children to act as change agents on weight, physical activity and diet of their mothers: a cluster randomized controlled trial | Gunawardena | 2016 | South Asia | Adults |
|  | Effects of controlled school-based multi-component model of nutrition and lifestyle interventions on behavior modification, anthropometry and metabolic risk profile of urban Asian Indian adolescents in North India | Singhal | 2010 | South Asia | Older children/adolescents |
|  | Impact of School-Based Health Behavioral Intervention on Awareness, Practice Pattern of Healthy Lifestyle, and Cardiometabolic Risk Factors among School Children of Shimla: A Cluster-Randomized, Intervention Study | Mahajan | 2022 | South Asia | Older children/adolescents |
|  | Effectiveness of information technology-enabled 'SMART Eating' health promotion intervention: A cluster randomized controlled trial | Kaur | 2020 | South Asia | Adults |
|  | Changing household dietary behaviours through community-based networks: A pragmatic cluster randomized controlled trial in rural Kerala, India | Daivadanam | 2018 | South Asia | Adults |
|  | Did HealthKick, a randomised controlled trial primary school nutrition intervention improve dietary quality of children in low-income settings in South Africa? | Steyn | 2015 | Sub Saharan Africa | Older children/adolescents |
|  | A Pre and Post Survey to Determine Effectiveness of a Dietitian-Based Nutrition Education Strategy on Fruit and Vegetable Intake and Energy Intake among Adults | Pem | 2016 | Sub Saharan Africa | Adults |
|  | Ultra-processed Food Consumption by Pregnant Women: The Effect of an Educational Intervention with Health Professionals | Gomes | 2019 | Latin America and Caribbean | Adults |
|  | Maternal Dietary Counseling Reduces Consumption of Energy-Dense Foods among Infants: A Randomized Controlled Trial | Vitolo | 2012 | Latin America and Caribbean | Adults |
|  | Improving Nutrition Habits and Reducing Sedentary Time Among Preschool-Aged Children in Cuenca, Ecuador: A Trial of a School-Based Intervention | Romo | 2018 | Latin America and Caribbean | Pre-school children |
|  | Effectiveness of the VAMOS Strategy for Increasing Physical Activity and Healthy Dietary Habits: A Randomized Controlled Community Trial | Meurer | 2019 | Latin America and Caribbean | Adults |
|  | Effectiveness of an educational intervention to reduce the consumption of high-calorie foods in public school children in Teresina, Piaua (Brazil) | Melo dos Santos | 2015 | Latin America and Caribbean | Older children/adolescents |
|  | The effects of a school-based intervention programme on dietary intakes and physical activity among primary-school children in Trinidad and Tobago | Francis | 2010 | Latin America and Caribbean | Older children/adolescents |
|  | Effects of a nutritional intervention using pictorial representations for promoting knowledge and practices of healthy eating among Brazilian adolescents | Fonseca | 2019 | Latin America and Caribbean | Older children/adolescents |
|  | Evaluating the effect of nutritional education on the prevalence of overweight/obesity and on foods eaten at primary schools | Fernandes | 2009 | Latin America and Caribbean | Older children/adolescents |
|  | Evaluation of the Computer-Based Intervention Program Staying fit Brazil to Promote Healthy Eating Habits: The Results from a School Cluster-Randomized Controlled Trial | da Silva | 2019 | Latin America and Caribbean | Older children/adolescents |
|  | Effectiveness of a Randomized School-Based Intervention Involving Families and Teachers to Prevent Excessive Weight Gain among Adolescents in Brazil | Cunha | 2013 | Latin America and Caribbean | Older children/adolescents |
|  | Effects of the Rango Cards game intervention on food consumption, nutritional knowledge and self-efficacy in the adoption of healthy eating practices of high school students: a cluster randomised controlled trial | Chagas | 2020 | Latin America and Caribbean | Older children/adolescents |
|  | Improving Heart Healthy Lifestyles Among Participants in a Salud Para Su Corazon Promotores Model: The Mexican Pilot Study, 2009-2012 | Balcazar | 2015 | Latin America and Caribbean | Adults |
|  | Alteration in unhealthy nutrition behaviors in adolescents through community intervention: Isfahan Healthy Heart Program | Mohammadifard | 2013 | Middle East and North Africa | Older children/adolescents |
|  | Is an Iranian Health Promoting School status associated with improving school food environment and snacking behaviors in adolescents? | Yazdi-Feyzabadi | 2018 | Middle East and North Africa | Older children/adolescents |
|  | The effect of personal lifestyle intervention among health care providers on their patients and clinics; the Promoting Health by Self Experience (PHASE) randomized controlled intervention trial | Shai | 2012 | Middle East and North Africa | Adults |
|  | Using Social Marketing to Reduce Salt Intake in Iran | Layeghiasl | 2020 | Middle East and North Africa | Adults |
|  | Using Digital Platform Approach to Reduce Salt Intake in a Sample of UAE Population: An Intervention Study | Jarrar | 2022 | Middle East and North Africa | Adults |
|  | APPLE Project: 2-y findings of a community-based obesity prevention program in primary school aged children | Taylor | 2007 | East Asia and Pacific | Older children/adolescents |
|  | After-school nutrition education programme improves eating behaviour in economically disadvantaged adolescents | Shen | 2021 | East Asia and Pacific | Older children/adolescents |
|  | Effect of a low-intensity parent-focused nutrition intervention on dietary intake of 2-to 5-year olds | Duncanson | 2013 | East Asia and Pacific | Pre-school children |
|  | Effectiveness of a Singaporean Community-Based Physical Activity and Nutrition Intervention: A Cluster Randomized Controlled Trial | Wong | 2021 | East Asia and Pacific | Adults |
|  | The Role of a Food Literacy Intervention in Promoting Food Security and Food Literacy-OzHarvest's NEST Program | West | 2020 | East Asia and Pacific | Adults |
|  | Does education level affect the efficacy of a community based salt reduction program? - A post-hoc analysis of the China Rural Health Initiative Sodium Reduction Study (CRHI-SRS) | Wang | 2016 | East Asia and Pacific | Adults |
|  | A parent-based intervention to promote healthy eating and active behaviours in pre-school children: evaluation of the MEND 2-4 randomized controlled trial | Skouteris | 2016 | East Asia and Pacific | Pre-school children |
|  | Maternal nutrition intervention focused on the adjustment of salt and sugar intake can improve pregnancy outcomes | Seo | 2020 | East Asia and Pacific | Adults |
|  | Social robots as treatment agents: Pilot randomized controlled trial to deliver a behavior change intervention | Robinson | 2020 | East Asia and Pacific | Adults |
|  | Assessment of a Salt Reduction Intervention on Adult Population Salt Intake in Fiji | Pillay | 2017 | East Asia and Pacific | Adults |
|  | Improving diet, physical activity and other lifestyle behaviours using computer-tailored advice in general practice: a randomised controlled trial | Parekh | 2012 | East Asia and Pacific | Adults |
|  | Evidence-Based Nutrition Interventions Improved Adolescents' Knowledge and Behaviors in Indonesia | Oddo | 2022 | East Asia and Pacific | Older children/adolescents |
|  | Evaluation of the Good Start Program: a healthy eating and physical activity intervention for Maori and Pacific Islander children living in Queensland, Australia | Mihrshahi | 2017 | East Asia and Pacific | Older children/adolescents |
|  | Effects of Integrating Pedometers, Parental Materials, and E-mail Support Within an Extracurricular School Sport Intervention | Lubans | 2009 | East Asia and Pacific | Older children/adolescents |
|  | A parent focused child obesity prevention intervention improves some mother obesity risk behaviors: the Melbourne Infant program | Lioret | 2012 | East Asia and Pacific | Pre-school children; Adults |
|  | Behavioral intervention reduces unhealthy eating behaviors in preschool children via a behavior card approach | Lin | 2016 | East Asia and Pacific | Pre-school children |
|  | The Chirpy Dragon intervention in preventing obesity in Chinese primary-school--aged children: A cluster-randomised controlled trial | Li | 2019 | East Asia and Pacific | Older children/adolescents |
|  | Digital Education to Limit Salt in the Home (DELISH) Program Improves Knowledge, Self-Efficacy, and Behaviors Among Children | Grimes | 2018 | East Asia and Pacific | Older children/adolescents |
|  | Evaluation of Fast Food Behavior in Pre-School Children and Parents Following a One-Year Intervention with Nutrition Education | Gao | 2014 | East Asia and Pacific | Pre-school children; Older children/adolescents; Adults |
|  | Evaluation of a computer-tailored lifestyle modification support tool for employees in Japan | Fuji | 2009 | East Asia and Pacific | Adults |
|  | Evaluation of a peer education program on student leaders' energy balance-related behaviors | Foley | 2017 | East Asia and Pacific | Older children/adolescents |
|  | Physical activity and nutrition programs for couples: a randomized controlled trial | Burke | 2003 | East Asia and Pacific | Adults |
|  | Dietary Outcomes of the 'Healthy Youngsters, Healthy Dads' Randomised Controlled Trial | Ashton | 2021 | East Asia and Pacific | Pre-school children; Adults |
|  | The effects of a 2-year individualized and family-based lifestyle intervention on physical activity, sedentary behavior and diet in children | Viitasalo | 2016 | Europe and Central Asia | Older children/adolescents |
|  | Effects of an intervention aimed at reducing the intake of sugar-sweetened beverages in primary school children: a controlled trial | vandeGaar | 2014 | Europe and Central Asia | Older children/adolescents |
|  | Effectiveness of a universal parental support programme to promote healthy dietary habits and physical activity and to prevent overweight and obesity in 6-year-old children: the healthy school start study, a cluster-randomised controlled trial | Nyberg | 2015 | Europe and Central Asia | Older children/adolescents; Adults |
|  | Preventing childhood obesity by reducing consumption of carbonated drinks: cluster randomised controlled trial | James | 2004 | Europe and Central Asia | Older children/adolescents |
|  | The effects of a middle-school healthy eating intervention on adolescents' fat and fruit intake and soft drinks consumption | Haerens | 2007 | Europe and Central Asia | Older children/adolescents |
|  | Changes in adolescents' intake of sugar-sweetened beverages and sedentary behaviour: results at 8 month mid-way assessment of the HEIA study-a comprehensive, multi-component school-based randomized trial | Bjelland | 2011 | Europe and Central Asia | Older children/adolescents |
|  | Impact of a nutrition education programme on free sugar intake & nutrition-related knowledge in fifth-grade schoolchildren | Winzer | 2021 | Europe and Central Asia | Older children/adolescents |
|  | The Viennese EDDY Study as a Role Model for Obesity: Prevention by Means of Nutritional and Lifestyle Interventions | Widhalm | 2018 | Europe and Central Asia | Older children/adolescents |
|  | A nutritional intervention programme at a worksite canteen to promote a healthful lifestyle inspired by the traditional Mediterranean diet | Vitale | 2018 | Europe and Central Asia | Adults |
|  | Effectiveness of a Worksite Intervention for Male Construction Workers on Dietary and Physical Activity Behaviors, Body Mass Index, and Health Outcomes: Results of a Randomized Controlled Trial | Viester | 2018 | Europe and Central Asia | Adults |
|  | Children Learn, Children Do! Results of the "Planning Health in School", a Behavioural Change Programme | Vieira | 2021 | Europe and Central Asia | Older children/adolescents |
|  | The impact of a nutrition education intervention on main meal quality and fruit intake in people with financial problems | vanAssema | 2005 | Europe and Central Asia | Adults |
|  | Impact of a youth-led social marketing intervention run by adolescents to encourage healthy lifestyles among younger school peers (EYTO-Kids project): a parallel-cluster randomised controlled pilot study | Tarro | 2019 | Europe and Central Asia | Older children/adolescents |
|  | Development and preliminary evaluation of an internet-based healthy eating program: randomized controlled trial | Tapper | 2014 | Europe and Central Asia | Adults |
|  | Effects of 1 y of free school fruit on intake of fruits, vegetables, and unhealthy snacks: 14 y later | Stea | 2018 | Europe and Central Asia | Adults |
|  | Short- and Medium-Term Efficacy of a Web-Based Computer-Tailored Nutrition Education Intervention for Adults Including Cognitive and Environmental Feedback: Randomized Controlled Trial | Springvloet | 2015 | Europe and Central Asia | Adults |
|  | Effects of the Healthy Start randomized intervention on dietary intake among obesity-prone normal-weight children | Rohde | 2017 | Europe and Central Asia | Pre-school children; Older children/adolescents |
|  | Impact of a sodium-reduced bread intervention with and without dietary counseling on sodium intake-a cluster randomized controlled trial among Danish families | Riis | 2020 | Europe and Central Asia | Pre-school children; Older children/adolescents; Adults |
|  | The Effectiveness of a Smartphone Application on Modifying the Intakes of Macro and Micronutrients in Primary Care: A Randomized Controlled Trial. The EVIDENT II Study | Recio-Rodriguez | 2018 | Europe and Central Asia | Adults |
|  | Neurological development of 5-year-old children receiving a low-saturated fat, low-cholesterol diet since infancy: A randomized controlled trial | Rask-Nissila | 2000 | Europe and Central Asia | Pre-school children |
|  | Nutrition knowledge and food intake of seven-year-old children in an atherosclerosis prevention project with onset in infancy: the impact of child-targeted nutrition counselling given to the parents | Rasanen | 2021 | Europe and Central Asia | Older children/adolescents |
|  | Family dietary coaching to improve nutritional intakes and body weight control: a randomized controlled trial | Paineau | 2008 | Europe and Central Asia | Older children/adolescents; Adults |
|  | Efficacy and use of an internet-delivered computer-tailored lifestyle intervention, targeting saturated fat intake, physical activity and smoking cessation: a randomized controlled trial | Oenema | 2008 | Europe and Central Asia | Adults |
|  | Mobile-based intervention intended to stop obesity in preschool-aged children: the MINISTOP randomized controlled trial | Nystrom | 2017 | Europe and Central Asia | Pre-school children |
|  | Compensation and transfer effects of eating behavior change in daily life: Evidence from a randomized controlled trial | Nigg | 2021 | Europe and Central Asia | Adults |
|  | Longitudinal effect of 20-year infancy-onset dietary intervention on food consumption and nutrient intake: the randomized controlled STRIP study | Matthews | 2019 | Europe and Central Asia | Pre-school children; Older children/adolescents; Adults |
|  | Krachtvoer: effect evaluation of a Dutch healthful diet promotion curriculum for lower vocational schools | Martens | 2008 | Europe and Central Asia | Older children/adolescents |
|  | Health and nutrition education in primary schools of Crete: changes in chronic disease risk factors following a 6-year intervention programme | Manios | 2002 | Europe and Central Asia | Older children/adolescents |
|  | Effect of Dietary Counseling on a Comprehensive Metabolic Profile from Childhood to Adulthood | Lehtovirta | 2018 | Europe and Central Asia | Pre-school children; Older children/adolescents; Adults |
|  | The efficacy of Web-based and print-delivered computer-tailored interventions to reduce fat intake: results of a randomized, controlled trial | Kroeze | 2008 | Europe and Central Asia | Adults |
|  | Comparing Diet and Exercise Monitoring Using Smartphone App and Paper Diary: A Two-Phase Intervention Study | Jimoh | 2018 | Europe and Central Asia | Older children/adolescents |
|  | Effect of a tailored behavior change program on a composite lifestyle change score: a randomized controlled trial | Jacobs | 2011 | Europe and Central Asia | Adults |
|  | Using Smartphone-Based Support Groups to Promote Healthy Eating in Daily Life: A Randomised Trial | Inauen | 2017 | Europe and Central Asia | Adults |
|  | The diet of adolescents can be improved by school intervention | Hoppu | 2010 | Europe and Central Asia | Older children/adolescents |
|  | Short-Term Pilot Study to Evaluate the Impact of Salbi Educa Nutrition App in Macronutrients Intake and Adherence to the Mediterranean Diet: Randomized Controlled Trial | Gonzalez-Ramirez | 2022 | Europe and Central Asia | Adults |
|  | The effect of complex workplace dietary interventions on employees' dietary intakes, nutrition knowledge and health status: a cluster controlled trial | Geaney | 2016 | Europe and Central Asia | Adults |
|  | The Effect of a Serious Health Game on Children's Eating Behavior: Cluster-Randomized Controlled Trial | Folkvord | 2021 | Europe and Central Asia | Older children/adolescents |
|  | Association between a school-based intervention and adiposity outcomes in adolescents: The Italian "EAT" project | Ermetici | 2016 | Europe and Central Asia | Older children/adolescents |
|  | Tailoring dietary feedback to reduce fat intake: an intervention at the family level | De Bourdeaudhuij | 2000 | Europe and Central Asia | Older children/adolescents; Adults |
|  | The Effects of a Computer Game (Healthy Rat King) on Preschool Children's Nutritional Knowledge and Junk Food Intake Behavior: Nonrandomized Controlled Trial | Chang | 2022 | Europe and Central Asia | Pre-school children |
|  | PEGASO e-Diary: User Engagement and Dietary Behavior Change of a Mobile Food Record for Adolescents | Caon | 2022 | Europe and Central Asia | Older children/adolescents |
|  | Healthier food choices as a result of the revised healthy diet programme Krachtvoer for students of prevocational schools | Bessems | 2012 | Europe and Central Asia | Older children/adolescents |
|  | Efficacy of a minimal intervention to reduce fat intake | Armitage | 2001 | Europe and Central Asia | Adults |
|  | Text Messages to Curb Sugar-Sweetened Beverage Consumption among Pregnant Women and Mothers: A Mobile Health Randomized Controlled Trial | Woo Baidal | 2021 | North America | Adults |
|  | Piloting “sodabriety”: a school-based intervention to impact sugar-sweetened beverage consumption in rural Appalachian high schools | Smith | 2014 | North America | Older children/adolescents |
|  | Mobile technology for obesity prevention: a randomized pilot study in racial-and ethnic-minority girls | Nollen | 2014 | North America | Older children/adolescents |
|  | Effects of interventions based on the theory of planned behavior on sugar-sweetened beverage consumption intentions and behavior | Gregorio-Pascual | 2020 | North America | Adults |
|  | A multicomponent intervention helped reduce sugar-sweetened beverage intake in economically disadvantaged Hispanic children | Feng | 2016 | North America | Older children/adolescents |
|  | Childhood obesity prevention and control in city recreation centres and family homes: the MOVE/me Muevo Project | Elder | 2014 | North America | Older children/adolescents |
|  | Effects of a behavioral and health literacy intervention to reduce sugar-sweetened beverages: a randomized-controlled trial | Zoellner | 2016 | North America | Adults |
|  | Development and Pilot Testing of Text Messages to Help Reduce Sugar-Sweetened Beverage Intake Among Rural Caregivers and Adolescents: Mixed Methods Study | Yuhas | 2019 | North America | Older children/adolescents; Adults |
|  | "Healthy-start": outcome of an intervention to promote a heart healthy diet in preschool children | Williams | 2002 | North America | Pre-school children |
|  | Does nutrition information on menus impact food choice? Comparisons across two hospital cafeterias | Vanderlee | 2014 | North America | Adults |
|  | A multilevel, multicomponent childhood obesity prevention group-randomized controlled trial improves healthier food purchasing and reduces sweet-snack consumption among low-income African-American youth | Trude | 2018 | North America | Older children/adolescents |
|  | An Electronic Wellness Program to Improve Diet and Exercise in College Students: A Pilot Study | Schweitzer | 2016 | North America | Adults |
|  | A community-based, culturally relevant intervention to promote healthy eating and physical activity among middle-aged African American women in rural Alabama: findings from a group randomized controlled trial | Scarinci | 2014 | North America | Adults |
|  | A Cluster-Randomized Controlled Trial to Evaluate a Community-Based Healthy Eating and Nutrition Label Interpretation Intervention Among Latinx Immigrant Mothers and Their Daughters | Scarinci | 2020 | North America | Older children/adolescents; Adults |
|  | Learning to health yourself: a randomized, tailored self-regulation intervention among custodial employees | Rameshbabu | 2018 | North America | Adults |
|  | Effectiveness of employee internet-based weight management program | Petersen | 2008 | North America | Adults |
|  | Weight Gain Prevention among Midlife Women: A Randomized Controlled Trial to Address Needs Related to the Physical and Social Environment | Perry | 2016 | North America | Adults |
|  | Randomized controlled trial of a primary care and home-based intervention for physical activity and nutrition behaviors: PACE+ for adolescents | Patrick | 2006 | North America | Older children/adolescents |
|  | Assessing Physical Activity, Fruit, Vegetable, and Sugar-Sweetened Beverage Intake Patterns of College Students in Kansas | Opoku-Acheampong | 2018 | North America | Adults |
|  | An environmental intervention to prevent excess weight gain in African-American students: a pilot study | Newton | 2010 | North America | Older children/adolescents |
|  | An Implementation Approach Comparison of a Child Care Center-Based Obesity Prevention Program | Natale | 2021 | North America | Pre-school children; Adults |
|  | Incorporating prototyping and iteration into intervention development: a case study of a dining hall-based intervention | McClain | 2013 | North America | Adults |
|  | Using a Community-Based Participatory Mixed Methods Research Approach to Develop, Evaluate, and Refine a Nutrition Intervention to Replace Sugary Drinks with Filtered Tap Water among Predominantly Central-American Immigrant Families with Infants and Toddlers: The Water Up @Home Pilot Evaluation Study | McCarley | 2021 | North America | Adults |
|  | Impact of the Out-of-School Nutrition and Physical Activity (OSNAP) Group Randomized Controlled Trial on Children's Food, Beverage, and Calorie Consumption among Snacks Served | Lee | 2018 | North America | Older children/adolescents |
|  | RE-AIM Analysis of a School-Based Nutrition Education Intervention in Kindergarteners | Larsen | 2017 | North America | Pre-school children |
|  | Kids SIP smartER: A Feasibility Study to Reduce Sugar-Sweetened Beverage Consumption Among Middle School Youth in Central Appalachia | Lane | 2018 | North America | Older children/adolescents |
|  | Impact of a School-Based Gardening, Cooking, Nutrition Intervention on Diet Intake and Quality: The TX Sprouts Randomized Controlled Trial | Landry | 2021 | North America | Older children/adolescents |
|  | The effects of Young Adults Eating and Active for Health (YEAH): a theory-based Web-delivered intervention | Kattelmann | 2014 | North America | Adults |
|  | A Habit-Based Randomised Controlled Trial to Reduce Sugar-Sweetened Beverage Consumption: the Impact of the Substituted Beverage on Behaviour and Habit Strength | Judah | 2020 | North America | Adults |
|  | Improving diet and exercise in pregnancy with Video Doctor counseling: a randomized trial | Jackson | 2011 | North America | Adults |
|  | Effects of lifestyle intervention on dietary intake, physical activity level, and gestational weight gain in pregnant women with different pre-pregnancy Body Mass Index in a randomized control trial | Hui | 2014 | North America | Adults |
|  | Targeting children's dietary behaviors in a family intervention: 'Entre familia: reflejos de salud' | Horton | 2013 | North America | Older children/adolescents |
|  | The effects of a family fitness program on the physical activity and nutrition behaviors of third-grade children | Hopper | 2015 | North America | Older children/adolescents |
|  | Tu Salud! Si Cuenta! Your Health Matters! A Community-wide Campaign in a Hispanic Border Community in Texas | Heredia | 2017 | North America | Adults |
|  | Six-week Latino family prevention pilot program effectively promotes healthy behaviors and reduces obesogenic behaviors | Hammons | 2013 | North America | Older children/adolescents; Adults |
|  | A group randomized controlled trial integrating obesity prevention and control for postpartum adolescents in a home visiting program | Haire-Joshu | 2015 | North America | Older children/adolescents |
|  | Effect of a Nutrient Rich Foods consumer education program: results from the nutrition advice study | Glanz | 2012 | North America | Adults |
|  | A social marketing theory-based diet-education program for women ages 54 to 83 years improved dietary status | Francis | 2009 | North America | Adults |
|  | Changes in diet and physical activity resulting from the Shape Up Somerville community intervention | Folta | 2013 | North America | Older children/adolescents |
|  | Title: efficacy of a food parenting intervention for mothers with low income to reduce preschooler's solid fat and added sugar intakes: a randomized controlled trial | Fisher | 2019 | North America | Pre-school children |
|  | Long-term effects of a communication intervention for Spanish-dominant Latinas | Elder | 2006 | North America | Adults |
|  | The worksite heart health improvement project's impact on behavioral risk factors for cardiovascular disease in long-term care: A randomized control trial | Doran | 2018 | North America | Adults |
|  | Practice-based evidence of effectiveness in an integrated nutrition and parenting education intervention for low-income parents | Dickin | 2013 | North America | Adults |
|  | It's all about kids: preventing overweight in elementary school children in Tulsa, OK | DeVault | 2009 | North America | Older children/adolescents |
|  | Randomized trial of a "talking computer" to improve adults' eating habits | Delichatsios | 2001 | North America | Adults |
|  | A Novel Personalized Systems Nutrition Program Improves Dietary Patterns, Lifestyle Behaviors and Health-Related Outcomes: Results from the Habit Study | de Hoogh | 2021 | North America | Adults |
|  | Environmental changes to control obesity: a randomized controlled trial in manufacturing companies | Brehm | 2011 | North America | Adults |
|  | Ability of a mass media campaign to influence knowledge, attitudes, and behaviors about sugary drinks and obesity | Boles | 2014 | North America | Adults |
|  | Preliminary healthy eating outcomes of SNaX, a pilot community-based intervention for adolescents | Bogart | 2011 | North America | Older children/adolescents |
|  | A culturally appropriate intervention to improve health behaviors in Hispanic mother-child dyads | Bender | 2013 | North America | Pre-school children; Adults |
|  | Reducing Obesogenic Eating Behaviors in Hispanic Children through a Family-Based, Culturally-Tailored RCT: Abriendo Caminos | Barragan | 2022 | North America | Older children/adolescents |
|  | Padres Preparados, Jovenes Saludables: intervention impact of a randomized controlled trial on Latino father and adolescent energy balance-related behaviors | Baltaci | 2022 | North America | Older children/adolescents; Adults |
|  | Evaluation of Online and In-Person Nutrition Education Related to Salt Knowledge and Behaviors among Special Supplemental Nutrition Program for Women, Infants, and Children Participants | Au | 2017 | North America | Adults |
|  | Latina mothers as agents of change in children's eating habits: findings from the randomized controlled trial Entre Familia: Reflejos de Salud | Arrendondo | 2018 | North America | Older children/adolescents; Adults |
|  | Nutrition education worksite intervention for university staff: application of the health belief model | Abood | 2003 | North America | Adults |
